# MYH9 Key Amino Acid Residues Identified by the Anti-Idiotypic Antibody to Porcine Reproductive and Respiratory Syndrome Virus Glycoprotein 5 Involve in the Virus Internalization by Porcine Alveolar Macrophages

**DOI:** 10.3390/v12010040

**Published:** 2019-12-29

**Authors:** Liangliang Li, Lu Zhang, Qifan Hu, Liang Zhao, Yuchen Nan, Gaopeng Hou, Yiyang Chen, Ximeng Han, Xiaolei Ren, Qin Zhao, Hu Tao, Zhenzhao Sun, Gaiping Zhang, Chunyan Wu, Jingfei Wang, En-Min Zhou

**Affiliations:** 1 Department of Preventive Veterinary Medicine, College of Veterinary Medicine, Northwest A&F University, Yangling 712100, China; lifeiyang2007@126.com (L.L.); zhanglunlzf@163.com (L.Z.); nanyuchen2015@nwsuaf.edu.cn (Y.N.); alexh2015@126.com (G.H.); chenyiyang0823@163.com (Y.C.); renxiaoleicn@foxmail.com (X.R.); qinzhao_2004@nwsuaf.edu.cn (Q.Z.); 2 College of Agronomy, Liaocheng University, Liaocheng 252059, China; 3 College of Life Science, Northwest A&F University, Yangling 712100, China; taohu@nwsuaf.edu.cn; 4 Harbin Veterinary Research Institute, Chinese Academy of Agricultural Sciences, Harbin 100193, China; 5 College of Animal Science and Veterinary Medicine, Henan Agricultural University, Zhengzhou 450002, China; zhanggaiping2003@163.com

**Keywords:** MYH9, anti-idiotype, PRRSV, PRRSV GP5, pathogenesis

## Abstract

MYH9 has been identified as an indispensable cellular protein for porcine reproductive and respiratory syndrome virus (PRRSV) entry into permissive cells using the monoclonal anti-idiotypic antibody (Mab2-5G2) recognizing an antibody that specifically interacts with PRRSV glycoprotein 5 (GP5). More recently, we found that Mab2-5G2 interacted with the MYH9 C-terminal domain, designated PRA, which is required for PRRSV internalization. In this study, we demonstrate that blocking of MYH9 with Mab2-5G2 significantly diminished PRRSV internalization by porcine alveolar macrophage (PAM) via interruption of direct interaction between GP5 and MYH9, and thus remarkably inhibited subsequent infection of PAMs by PRRSV-2 isolates. Moreover, the three-dimensional structure of the Mab2-5G2 Fab-PRA complex determined via homology modeling predicted potential docking sites required for PRRSV internalization. Further analysis of Mab2-5G2-binding sites within PRA highlighted that the amino acids E1670, K1673, E1679, and I1683 in PRA are the key Mab2-5G2-binding residues. Notably, recombinant PRA protein blocked the interaction between PRRSV GP5 and cellular MYH9 by preventing translocation of MYH9 from the cytoplasm to the cell membrane, an essential step for PRRSV virion internalization. Meanwhile, porcine cell line permissive for PRRSV bearing point mutation of E1670A in MYH9 demonstrated reduced susceptibility for PRRSV infection. In conclusion, this work increases understanding of both PRRSV pathogenesis and the mechanistic role played by MYH9 in PRRSV infection.

## 1. Introduction

Porcine reproductive and respiratory syndrome virus (PRRSV) is a positive-sense, single-stranded enveloped RNA virus belonging to the family *Arteriviridae*. All PRRSV strains have been categorized into two species within the genus *Porartevirus*, designated PRRSV-1 and PRRSV-2 [1,2]. *PRRSV-1* and *PRRSV-2* share approximately 60% nucleotide sequence identity and exhibit serotype differences [3,4]. PRRSV infection is highly restricted to cells of the monocyte-macrophage lineage in vivo, such as porcine alveolar macrophages (PAMs) [5,6].

Numerous studies demonstrate that PRRSV infection is mediated by various cellular receptors or factors [7], such as heparin sulfate (HS) [8], vimentin [9], CD151 [10], porcine CD163 (CD163) [11], sialoadhesin (CD169) [12], and DC-SIGN (CD209) [13]. Our previous studies demonstrated that an anti-idiotypic monoclonal antibody (Mab2-5G2) developed against antibodies to PRRSV-GP5 recognizes the C-terminal domain of MYH9 (hereafter designated PRA) present in PRRSV-permissive cells. Further analysis demonstrated that direct interaction between CD163 N-terminal domain and MYH9 C-terminal PRA domain contributes to PRRSV internalization by permissive cells [14]. Moreover, our latest research also indicated that the PRRSV-GP5 ectodomain interacts with MYH9 to induce MYH9 aggregation [15], a key process allowing myosin filament assembly and acquisition of motor activity [16,17], which facilitates entry of larger virus particles by bending internal and external membranes to enable internalization [18,19,20]. Therefore, it appears that MYH9 serves as a key host factor during PRRSV internalization into host cells [14,21,22].

According to the idiotypic network theory proposed by Jerne [23], anti-idiotypic antibodies can mimic the original antigen. Thus, anti-idiotypic antibodies mimicking viral antigen may be used as vaccine candidates to prime or stimulate the immune response against virus infection [24,25,26,27] or used as tools to identify virus receptor in permissive cells [28,29,30]. In our previous research, Mab2-5G2 was shown to react with cellular MYH9 protein from PRRSV-permissive cells [21]. MYH9 has been identified as a cellular receptor for herpes simplex virus-1 (HSV-1) [31], severe fever with thrombocytopenia syndrome virus (SFTSV) [32], Epstein-Barr virus (EBV), and PRRSV [21,33]. Regarding PRRSV, the PRA domain located within the C-terminal portion of MYH9 is responsible for binding to viral GP5, as demonstrated using a recombinant soluble form of PRA that blocked PRRSV infection in vitro [34,35].

In this study, we determined whether Mab2-5G2 blocked PRRSV infection in PAMs and characterized key amino acids of PRA domain that are responsible for Mab2-5G2 recognition. Notably, application of 3D homology modeling predicted potential docking sites (E1670, K1673, E1679, and I1683 of MYH9) was required for the interaction between Mab2-5G2 and PRA. Moreover, our preliminary data suggested that introduction of E1670A into wild-type MYH9 reduced the susceptibility of permissive cells to PRRSV infection, which provides valuable insight for understanding PRRSV–host interaction.

## 2. Material and Methods

### 2.1. Cells, Viruses, and Chemicals

MARC-145 cells and HEK-293T cells were maintained in Dulbecco’s Modified Eagle Medium (DMEM) containing 10% fetal bovine serum (FBS) (Hyclone, Chicago, IL, USA) supplemented with antibiotics (100 µg/mL each of streptomycin and ampicillin). Porcine alveolar macrophages (PAMs) were collected from a 4-weeks-old PRRSV-negative pig as previously described [34] and maintained in RPMI 1640 medium (Biological Industries, Beit HaEmek, Israel) supplemented with 10% FBS (Biological Industries). Hybridoma cells secreting Mab2-5G2 were made in-house and maintained in the same condition as PAMs.

PRRSV viruses used in this study included PRRSV-1 strain GZ11-G1 (GenBank: KF001144.1) and PRRSV-2 strains VR2385 (GenBank: JX044140.1), VR-2332 (GenBank: EF536003.1), SD16 (GenBank: JX087437.1), JXA1 (GenBank: EF112445.1), and GD-HD (GenBank: KP793736.1). Viruses were maintained in-house and used to inoculate MARC-145 cells or PAMs at the indicated multiplicity of infection (MOI) determined by evaluation of the median tissue culture infectious dose (TCID_50_). Titration of different PRRSV strains was conducted in MARC-145 cells as previously described [35].

### 2.2. Plasmid Construction and Transfection

Total RNA was extracted from cells using TRIzol Reagent (Thermo Fisher Scientific, Waltham, MA, USA) in accordance with the manufacturer’s instructions. Reverse transcription of RNA samples was conducted using a PrimeScript RT reagent Kit (TaKaRa, Dalian, China) as previously described (24, 34). Sequencing of Mab2-5G2-encoding sequences within cDNA obtained from hybridoma cells was conducted by Genewiz Co., Ltd. (Nanjing, China). All transfection of plasmids into mammalian cells was conducted using FuGENE^®^ HD Transfection Reagent (Promega, Madison, WI, USA) according to instruction by manufacturer.

### 2.3. Western Blot Analysis

Cell samples were harvested using NP-40 cell lysis buffer 24 h post-PRRSV inoculation unless noted otherwise. Protein concentrations were determined using the Pierce BCA Protein Assay Kit (Thermo Fisher Scientific) and samples were mixed with 5× SDS sample loading buffer before samples containing equal amounts of total protein were loaded onto 12% SDS-PAGE gels and separated proteins were transferred onto PVDF membranes as described previously [36]. Membranes were probed with Mab2-5G2 or 6D10 (against the PRRSV-N protein) or anti-β-actin mAb (Abcam, Cambridge, MA, USA). Specific binding of antibodies to targets was detected using horseradish peroxidase (HRP)-conjugated goat anti-mouse secondary antibody (Jackson ImmunoResearch, West Grove, PA, USA) and visualized using ECL substrate (Beyotime, Beijing, China).

### 2.4. Dot Blot Assay

MF1, MF2, MF3, MF4, and MF5 peptides of 95% purity were synthesized by GL Biochem Ltd. (Shanghai, China). The dot blot assay was conducted by directly applying peptides and full-length recombinant MF protein (produced from *E. coli* cells) onto nitrocellulose (NC) membranes within a dot blot apparatus, followed by blocking with 5% milk before incubation with Mab2-5G2 and HRP-conjugated goat anti-mouse secondary antibody (Jackson ImmunoResearch, West Grove, PA, USA).

### 2.5. Enzyme-Linked Immunosorbent Assay (ELISA)

To determine the binding of Mab2-5G2 to PRA, 96-well microplates (Corning, NY, USA) were coated with recombinant PRA protein at 4 °C overnight and blocked with 5% skim milk in PBST, then incubated with Mab2-5G2 antibody or anti-MYC antibody (isotype control) as primary antibody. Binding of PRA to Mab2-5G2 was detected using horseradish peroxidase (HRP)-conjugated goat anti-mouse IgG antibodies and visualized using a 3,3’,5,5’-tetramethylbenzidine (TMB) kit (TianGen, Beijing, China). Absorbances of individual wells were measured at a wavelength of 450 nm using a VictorTM X5 Multilabel Plate Reader (PerkinElmer, Waltham, MA, USA). To determine the binding of mutant PRA proteins to anti-PRA rabbit poly serum, 96-well microplates (Corning) were coated with recombinant PRA mutant proteins and incubated with rabbit anti-PRA polyclonal serum as the same protocol described above.

To determine the binding of mutant PRA proteins to PRRSV virions, purified PRRSV SD16 virions (7 × 107.875 plaque-forming units, PFU) were used to coat 96-well microplates overnight at 4 °C following by blocking with 5% skim milk in PBST. PRA mutant proteins (1 µg) were added to each well and incubated for 2 h at 37 °C. Wild-type PRA and PCV2 Cap proteins were included as positive or negative controls, respectively. Homemade rabbit anti-PRA polyclonal antibodies or mouse anti-His tag monoclonal antibody were used to evaluate binding of mutant PRA proteins to virions. The specific interaction was detected using HRP-conjugated goat anti-rabbit IgG or anti-mouse IgG antibodies and visualized using a TMB kit (TianGen). In addition, PRRSV particle capture assays were further conducted to evaluate the interaction between PRA mutants and the PRRSV virion as previously described [35].

### 2.6. Immunofluorescence Assays (IFAs)

PAMs were seeded onto coverslips in 24-well plates before receiving indicated treatments. Next, cells were fixed in 4% paraformaldehyde (Sigma-Aldrich, St. Louis, MO, USA) without membrane permeabilization. IFAs were carried out using Mab2-5G2 and convalescent serum from PRRSV infected pigs. Specific interactions between antibodies with their corresponding targets were detected by Alexa Fluor^®^ 488-labeled goat anti-mouse secondary antibody (Thermo Fisher Scientific) or Texas Red-labeled goat anti-swine secondary antibody (Jackson ImmunoResearch) [37]. Then, coverslips were mounted onto slides using ProLong^®^ Gold Antifade Reagent containing 4′,6-diamidino-2-phenylindole (DAPI) (Thermo Fisher Scientific) and observed under a confocal microscope (Leica Microsystems, Wetzlar, Germany). All images were captured and processed using Leica Application Suite X (Version 1.0., Leica Microsystems).

### 2.7. Cell Viability Assay

Cytotoxicities of Mab-5G2 and PRA mutant proteins were evaluated using the Cell Counting Kit-8 (CCK-8) (Beyotime) according to the manufacturer’s instructions. Briefly, cells were seeded into 96-well plates (1 × 105/well) and incubated with indicated treatments at 37 °C for 24 h. Next, CCK-8 reagent (10 µL/well) was added to cells followed by incubation for another 2 h. Viable cells were determined from absorbance measurements at 450 nm using a VictorTM X5 Multilabel Plate Reader (PerkinElmer).

### 2.8. Quantitative Real-Time PCR (qPCR)

Total RNA was extracted from cells using TRizol reagent (Invitrogen, Carlsbad, CA, USA) in accordance with the manufacturer’s instructions. Reverse transcription and qPCR were conducted using PrimeScript RT reagent Kit (TaKaRa) as previously described [36]. Transcripts of GAPDH were also amplified to normalize the total RNA input. Relative quantification of target genes was calculated using the 2−ΔΔCt method. Sequences of primers used for qPCR are listed in Table 1.

### 2.9. Homology Modeling and Circular Dichroism Spectroscopy

Protein secondary structures were evaluated by circular dichroism (CD) as follows: recombinant PRA was diluted to a final concentration of 50 mM in phosphate buffers of various salt concentrations or pH levels then incubated at 25 °C for 30 min. CD spectra were acquired using a Jasco spectropolarimeter (model J-815) using a bandwidth of 1 nm with 1-nm step resolution from 190 to 260 nm at room temperature. Spectra were corrected by subtraction of values obtained from a blank (solvent only). The α-helical content was calculated from the CD signal by dividing the mean residue ellipticity (θ) at 222 nm by the value expected for 100% helix formation (−33,000∙degree∙cm^2^∙dmol^−1^).

Homology models for Mab2-5G2 were created using SWISS-MODEL (https://www.swissmodel.expasy.org), and graphical displays were generated using Chimera software (http://www.cgl.ucsf.edu/chimera/, production release 1.13). After homology models were generated, molecular docking between Mab2-5G2 Fab and PRA was simulated using the Discovery Studio Predictive Science Application Suite (Version 2.5, Accelrys, San Diego, CA, USA).

### 2.10. PRRSV Inhibition Assay

PAMs were seeded into 24-well cell plates at a cell density of 1 × 106 cells/well. Twelve hours later, PAMs were either pretreated with the indicated dose of Mab2-5G2 or left untreated before PRRSV inoculation at a MOI of 0.1. One hour after PRRSV inoculation, unbound virus and antibodies were removed by washing PAMs with PBS and the medium was replaced with RPMI 1640 containing 3% FBS. PRRSV replication in PAMs was determined by evaluating the expression of PRRSV ORF-7 via qPCR and Western blot. For the antibody isotype control, 1.25 μM normal mouse IgG was used.

To evaluate the inhibitory effect of PRA mutants on PRRSV replication, mutated PRA or wild-type PRA proteins were mixed with PRRSV at indicated concentrations to create PRAs-virus mixtures and incubated at 37 °C for 1 h before adding the mixtures to MARC-145 or PAMs. After 1 h of incubation of cells with PRAs-virus mixtures, unbound PRRSV virions were removed by washing cells with fresh PBS three times before cells were incubated for another 24 h then subjected to further analysis.

### 2.11. Evaluation of Virus Attachment and Internalization

Analysis of virion attachment to PAMs was initiated using PAMs seeded in 6-well cell culture plates after PAMs had been pretreated with 6.25 or 12.5 µM of Mab2-5G2 or control mouse IgG for 1 h at 37 °C. Next, plates were chilled on ice before inoculation of wells with PRRSV SD16 (MOI = 10), then plates were incubated at 4 °C for 2 h to allow virion attachment to cells without triggering of virion internalization. Next, unbound virus was removed by five washes with cold PBS before cells were harvested using TRIzol (Thermo Fisher Scientific). The attached virus was quantified using qPCR to determine viral RNA.

To analyze virus internalization, PAMs were preincubated with 6.25 or 12.5 µM Mab2-5G2 and infected with PRRSV SD16 as described above. After changing the medium to 3% FBS RPMI 1640, PAMs were incubated at 37 °C for 1 h to trigger virion internalization via endocytosis. Next, uninternalized virions were removed by trypsin digestion then cells were harvested using TRIzol Reagent. Internalized virus was quantified using qPCR to measure viral RNA.

To understand the effect of PRA on virus attachment to PAMs and internalization, PAMs exposed to PRRSV-SD16 (MOI = 50) or SD16 preincubated with PRA were harvested as described above. PAMs exposed to PRRSV SD16 preincubated with Cap protein served as a negative control.

### 2.12. Expression of PRA, PRA Truncation, and PRA Mutants

Wild-type PRA and truncated PRA were each cloned into pCold-SUMO vector followed by expression and purification of their corresponding proteins as previously described [35]. Briefly, the pCold-SUMO-PRA plasmid was transformed into the *Escherichia coli* strain BL21 (DE3) and cultured in LB medium at 37 °C until induced with 0.5 mM isopropyl β-d-thiogalactoside (IPTG) at 15 °C. After IPTG induction, bacterial cells were collected and resuspended in cell lysis buffer (50 mM Tris–HCl (pH 7.5), 150 mM NaCl, 1 mM EDTA, 1 mM AEBSF (4-benzenesulfonyl fluoride hydrochloride), 5% glycerol) for sonication. Lysate cell debris was removed by centrifugation and SUMO-PRA in the supernatant was purified using Ni+ affinity chromatography (GE Healthcare, Uppsala, Sweden) with elution buffer (50 mM Tris (pH 7.5), 150 mM NaCl, 200mM imidazole). The fused SUMO tag was removed later by inoculating SUMO-PRA with recombinant Tobacco Etch Virus (rTEV) protease under 25 °C for 8 h with 1 mM Dithiothreitol (DTT) and 1mM EDTA as previously described [38], followed by a Strep-Tactin affinity chromatography (GE Healthcare) purification procedure according to the manufacturer’s instruction. Then PRA protein was further diluted with dialysis buffer (50 mM PB buffer (pH 7.5), 150 mM NaCl).

PRA mutants E1670A, K1673A, E1679A, and I1683A were generated using site-directed mutagenesis followed by cloning of mutated DNA into wild-type pCold-SUMO-PRA vector. Sequences of primers used for site-directed mutagenesis are listed in Table 1. All recombinant plasmid constructs were confirmed by DNA sequencing. PRA mutants were expressed using the same methods used for expression of wild-type PRA as described above.

### 2.13. Size-Exclusion Chromatography

The size-exclusion chromatography (SEC) was conducted to analyze the aggregation of wild-type PRA and PRA mutants. Briefly, Superose^TM^ 6 increase 10/300GL (GE healthcare) were connected to ÄKTA pure 25 chromatography system (GE healthcare) and balanced using elution buffer (0.1 mol NaCl, 0.05 mol NaH_2_PO_4_. 2H_2_O, pH 7.5) first. Next, 1 mg wild-type PRA and PRA mutants were inputted and eluted using same elution buffer with elution speed of 0.5 mL/min. The absorbances peaks (mAU 280 nm) for wild-type PRA and PRA mutants were monitored and recorded for further analysis.

### 2.14. Plasma Membrane Isolation

After indicated treatments, PAMs were harvested at indicated time points for plasma membrane isolation. Plasma membrane isolation was done using a MinuteTM Plasma Membrane Protein Isolation Kit (Invent Biotechnologies, Inc., Plymouth, MN, USA) according to the manufacturer’s instructions. Plasma membrane samples from indicated groups were further subjected to SDS-PAGE and Western blotting using Mab-5G2 as a probe to determine the MYH9 recruitment from cytoplasm to the plasma membrane.

### 2.15. Generation of PK-15 Cells Bearing Point Mutation in MYH9

PK-15 cells with stable expression of porcine CD163, which is susceptible to PRRSV infection, were previously described [39]. Single amine acid mutation of original Glu residue to Ala of aa1670 in MYH9 was introduced using to PK-15^CD163^ by CRISPR/CAS9 technology and conducted by Sino Biological Co Ltd. (Beijing, China). The gRNA sequence was: gpMYH9-1:5′GGAGATCCTGGCACAGGCCA’3 and gpMYH9-2 5′CAGCTTCTTCTCGTTCTCCT3′ with the targeting genome MYH9 DNA sequence (GenBank Accession: NC_010447.5) of 5′ CTGCAGCTGGATCATCTCGGCCTCCATGCTTTTCAGCTTCTTCTCGTTCgCCTTGGCCTGTGCCAGGATCTCCTCGCGGGAGGCACGCGTGTCCTCCAGC’3. The introduction of mutation was confirmed by DNA sequencing of cDNA reverse transcribed from of MYH9 mRNA extracted from PK-15^CD163-MYH9E1670A^ cells.

### 2.16. Statistical Analysis

Statistical analysis was performed using GraphPad Prism version 5.0 (GraphPad Software, San Diego, CA, USA). Differences in indicators between treatment groups and controls were assessed using the Student’s t-test. A two-tailed P-value of less than 0.05 was considered statistically significant.

## 3. Results

### 3.1. Mapping of Binding Sites of Mab2-5G2 for MYH9

**Figure 1:**
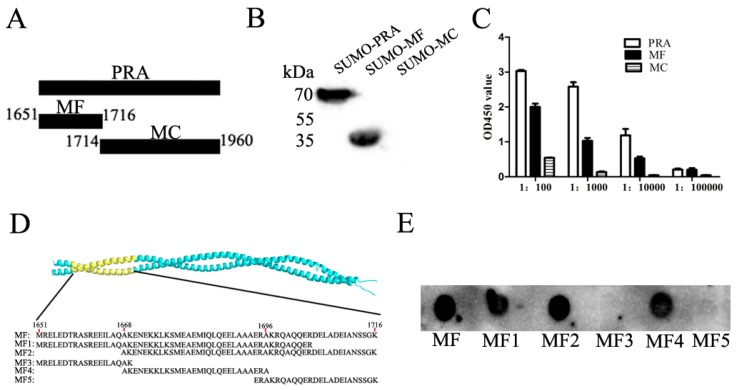
Determination of the binding site for Mab2-5G2 on MYH9. (**A**) Schematic illustration of PRA truncations: MF (aa 1651–1716) as the N-terminal of PRA and MC (aa 1714–1960) as the C-terminal of PRA. (**B**) Western blot analysis of PRA and truncated PRA fragments MF and MC using Mab2-5G2. (**C**) ELISA analysis of MF or MC binding to plate wells coated with Mab2-5G2 with PRA protein included as a positive control. ELISA data were presented as the mean ± SD. (**D**) Schematic illustration of peptide-binding locations mapped to the full-length MF fragment of the PRA region. (**E**) Dot blot assay using 5 µg of MF, MF1, MF2, MF3, MF4, and MF5 peptides applied to the blot and tested for binding to Mab2-5G2 as primary antibody.

Our previous studies demonstrated that an anti-idiotypic monoclonal antibody (Mab2-5G2) specific for an idiotypic antibody to PRRSV-GP5 recognizes the C-terminal domain of MYH9 (hereafter designated PRA), and PRA domain plays an indispensable role in PRRSV internalization into permissive cells [14,16,17]. Therefore, it is interesting to know the exact binding sites of Mab2-5G2 to a shorter stretch of amino acids within the PRA domain. Two truncated forms of PRA, MF (aa1651–1716), and MC (aa1714–1960) were produced and tested for their interactions with Mab2-5G2 (Figure 1A). As shown in Figure 1B, Mab2-5G2 only recognized the MF fragment, as determined via Western blot, and this result was consistent with ELISA results (Figure 1C) using purified SUMO-MF as coating antigens. To produce truncated MF fragments, five peptides, designated MF1–MF5 (Figure 1D), were synthesized for dot blot assays to more precisely localize the recognizing site of MYH9 by Mab2-5G2. Based on the results, Mab2-5G2 recognized MF1, MF2, and MF4, suggesting that the binding site of Mab2-5G2 for PRA resided within the aa1668-1696 region of full-length MYH9 (Figure 1E).

### 3.2. Molecular Modeling of the Interaction Region within the Mab2-5G2 Fab-PRA Complex

**Figure 2:**
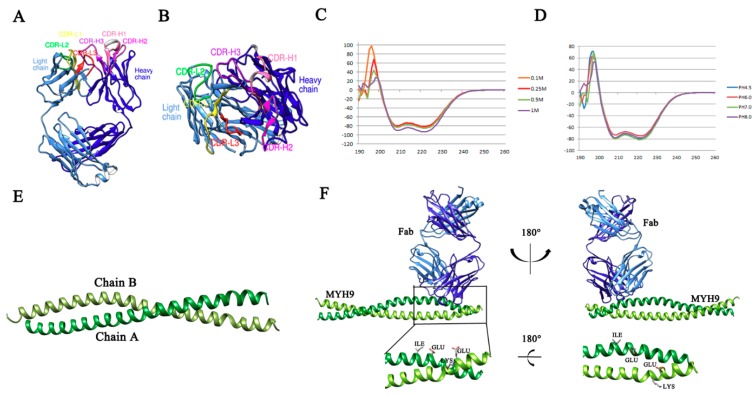
Structural basis of the predicted docking model of Mab2-5G2 Fab with its interacting region of MYH9. (**A**) Diagram showing a representation of the structure of Mab2-5G2 Fab via homology modeling. (**B**) Diagram showing a representation of the structure of Mab2-5G2 Fab via homology modeling of CDRs of Mab2-5G2. (**C**) CD spectra of native PRA protein structures in buffers containing various salt concentrations at pH 7.5. (**D**) CD spectra of native PRA protein structures in buffers with different pH values containing 150 mM NaCl. (**E**) Structure model of PRA domain within MYH9 with two α-chains, chain A shown in green and chain B shown in yellow. (**F**) The structure of predicted docking complex between Mab2-5G2 and its binding interaction region on MYH9.

Next, to understand the structural basis of the Mab2-5G2-PRA complex, we first characterized the complete cDNA sequence of the variable region of both heavy and light chains of Mab2-5G2 from corresponding hybridoma cells (Figure S1). Next, the three-dimensional (3D) structure of the Mab2-5G2 Fab was constructed using the SWISS-MODEL system for homology modeling as shown in Figure 2A. The structures of CDR-H1, CDR-H2, CDR-H3, CDR-L1, CDR-L2, and CDR-L3 are illustrated in Figure 2B. Conversely, Circular dichroism spectroscopy demonstrated that the PRA protein is primarily α-helical, as evidenced by characteristic minima at 222 and 208 nm observed in CD spectra of 50 μM PRA protein that was measured at 25 °C and pH 7.4 under different salt concentrations (Figure 2C) and in different buffers containing 150 mM NaCl with various pH values (Figure 2D). The C-terminal of MYH9 was comprised of α-helical coiled-coil, which is consistent with a previous report [40]. Based on homology modeling of the PRA 3D structure generated using the SWISS-MODE system (Figure 2E), the docking model between Mab2-5G2 Fab and PRA was further developed using Discovery Studio Client (version 2.5). Subsequently, four amino acids of PRA (Glu1670, Lys1673, Glu1679, and Ile1683) were predicted to be key residues required for Mab2-5G2 binding to MYH9, which may be involved in PRRSV internalization as well (Figure 2F).

### 3.3. Mab2-5G2 Blocks PRRSV Internalization by PAMs via Interaction with MYH9

**Figure 3:**
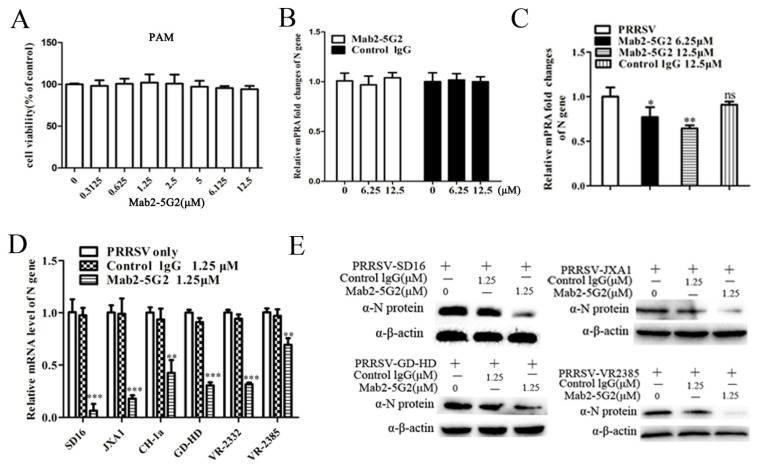
Mab2-5G2 treatment blocks porcine reproductive and respiratory syndrome virus (PRRSV) internalization. (**A**) Cell viability assay for cytotoxicity of Mab2-5G2 in porcine alveolar macrophages (PAMs). Gradient doses of Mab2-5G2 (0, 0.3125, 0.625, 1.25, 2.5, 5, 6.125, and 12.5 µM) were applied to PAMs for 24 h followed by viability testing of PAMs via Cell Counting Kit-8 (CCK-8) assay. No significant cytotoxicity was observed for Mab2-5G2. (**B**) PAMs were preincubated with Mab2-5G2 (6.25 and 12.5 µM) then followed by incubation of PRRSV-SD16 strain (10 MOI) under 4 °C for 2 h to allow sufficient attachment of virus, then PAMs from indicated groups were harvested for evaluating attached viral particles by qPCR analysis. PAMs treated with normal mouse IgG were included as isotype control. (**C**) PAMs were preincubated with Mab2-5G2 (6.25 and 12.5 µM) then followed by incubation of PRRSV-SD16 strain (10 MOI) under 4 °C for 2 h to allow sufficient attachment of virus, then the cells were shifted to 37 °C for 15 min and un-internalized PRRSV virions was removed by trypsin digestion. Samples were analyzed by relative quantification of internalized viral RNA by qPCR. All experiments were repeated at least three times. Significant differences between indicated groups are marked by “*” (*p* < 0.05) and “**” (*p* < 0.01). (**D**) PAMs were preincubated with Mab2-5G2 (1.25 µM) then followed by infection of different PRRSV-2 isolates (SD16, JXA1, CH-1a, GD-HD, VR2332 and VR-2385) for 24 h with a MOI of 0.1. Inhibitory activity of Mab2-5G2 on viral replication was determined by evaluation of relative fold of PRRSV-N gene expression using qPCR and compared to PRRSV infected PAMs without Mab2-5G2 treatment for each PRRSV strains. (**E**) PAMs were preincubated with Mab2-5G2 (1.25 µM) or left untreated followed by infection of different PRRSV-2 isolates (SD16, JXA1, GD-HD and VR-2385) for 24 h with a MOI of 0.1. Then expression of PRRSV N protein was examined by Western blot to further confirm the inhibitory effect of Mab2-5G2 against different PRRSV strains. All experiments were repeated at least three times. Significant differences between cells with or without Mab2-5G2 treatment are marked by “*” (*p* < 0.05), “**” (*p* < 0.01), and “***” (*p* < 0.001).

It has been demonstrated that MYH9 is required for PRRSV internalization and cell-to-cell spread among permissive cells [21,41]. To understand if the interaction of Mab2-5G2 and MYH9 would have an influence on PRRSV internalization into PAMs, we examined the levels of PRRSV replication in PAMs with or without Mab2-5G2. Based on the cytotoxicity assay (Figure 3A), the concentrations of 6.25 and 12.5 μM Mab2-5G2 were selected to investigate and internalized virions were determined via qPCR to evaluate PRRSV RNA level. Briefly, with pretreating PAMs with or without Mab2-5G2 or control mouse IgG, PAMs were further inoculated with PRRSV virions under 4 °C to allow sufficient virions attachment without virion internalization. By evaluating the attached virion via qPCR to quantify the viral RNA, our results demonstrated similar PRRSV-RNA levels among different treatment groups of PAMs, suggesting that interruption of the PRRSV virion interaction with MYH9 with Mab2-5G2 did not alter PRRSV virion attachment to PAMs (Figure 3B). However, if PAMs with indicated treatment groups incubated with PRRSV for 2 h at 4 °C were then shifted to 37 °C to trigger virion internalization, followed by removal of uninternalized virions by trypsin digestion before harvesting PAMs for qPCR, PRRSV RNA levels were significantly lower in PAMs receiving virus that had been pretreated with Mab2-5G2, which suggests that direct interaction between PRRSV-GP5 and MYH9 was needed for virion internalization (Figure 3C).

Conversely, we further examined if Mab2-5G2 inhibits the whole replication cycles of PRRSV in PAMs with pretreating PAMs prior to PRRSV inoculation. The results showed that Mab2-5G2 significantly inhibited heterogenous PRRSV strains replication in PAMs, as evidenced by a reduction of virus nucleocapsid (N) at both mRNA (Figure 3D) and protein levels (Figure 3E), while normal mouse lgG demonstrated minimum effect on PRRSV replication compared to PRRSV infected PAMs without treatment of any antibodies.

### 3.4. Interruption of Interaction between PRRSV-GP5 and MYH9 Blocked Internalization of PRRSV to Permissive Cells

As a cytoplasmic protein, it is known that MYH9 can relocate to the cell membrane and bind to viruses that utilize MYH9 as a receptor, such as HSV-1, EBV, and SFTSV [31,32]. Moreover, our previous study also demonstrated the direct interaction between PRRSV-GP5 and MYH9 induce aggregation of MYH9 to facilitate internalization of PRRSV virions [15]. Therefore, we wonder if interrupting the interaction between GP5 and MYH9 is capable to block internalization of PRRSV virions. To verify this, the recombinant PRA proteins, which have been previously shown to directly interact with GP5 from PRRSV virion, were used here as a decoy to block the interaction between PRRSV-GP5 and endogenous MYH9 from permissive cells.

**Figure 4:**
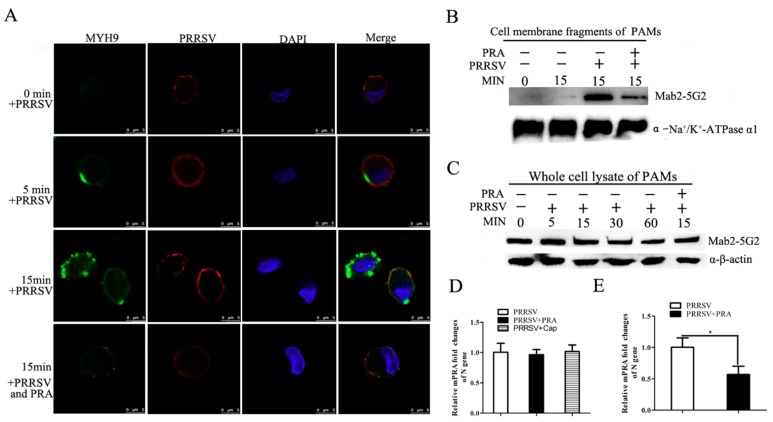
PRA treatment prevents both the redistribution of MYH9 to the plasma membrane and internalization of PRRSV virions. (**A**) Confocal microscopy of MYH9 redistribution from cytoplasm to membrane of PAMs with indicated treatments. PAMs were incubated with PRRSV (50 MOI) with or without preincubation with PRA (10 mg) at 4 °C for 2 h and then shifted to 37 °C. MYH9 redistribution from cytosol to plasma membrane was induced by PRRSV attachment to PAMs. All samples used here are not permeabilized by TritonX100 to maintain the integrity of plasma membrane. (**B**) PAMs were incubated with PRRSV (with or without preincubation with 10 mg PRA) with a MOI of 50 at 4 °C for 2 h followed by 15 min incubation after shifting to 37 °C, then plasma membrane fractions of PAMs from indicated groups were extracted and subjected to Western blot analysis using Mab2-5G2 for MYH9 detection. Plasma membrane protein Na^+^/K^+^-ATPase α1 was stained with corresponding antibodies as well and included as a sample-loading control. (**C**) PAMs were incubated with PRRSV at MOI = 50 at 4 °C for 2 h followed by shifting to 37 °C, whole-cell lysates of PAMs harvested at indicated time points (0, 5, 15, 30, 60 min) after temperature shifting were harvested and analyzed for total MYH9 expression via Western blot. PAMs incubated with PRRSV-PRA mixture (50 MOI and 10 mg, respectively) followed by 15 min incubation after shifting to 37 °C was included as well; (**D**) PAMs incubated with PRA-PRRSV virion mixture or PRRSV alone at 4 °C without internalization of virus were harvested using TRIzol for relative quantification of attached virions on cell surfaces via evaluating viral RNA by qPCR. (**E**) PAMs incubated with PRA-PRRSV virion mixture or MF4 peptide-PRRSV virion mixture at 4 °C were shifted to 37 °C to trigger internalization of PRRSV virions then followed by trypsin digestion to remove un-internalized virus; samples were harvested using TRIzol for relative quantification of internalized virus via evaluation of viral RNA by qPCR. PAMs incubated with PRRSV alone were included as positive control. The data are expressed as mean ± SD and were subjected to Student’s t-test. Significant differences between the two groups are marked by “*” (*p* < 0.05).

As shown in Figure S2, in normal cells with membrane permeabilization, endogenous MYH9 protein was even distributed in cytoplasm of uninfected PAMs. While in the non-permeabilization condition, after exposing PAMs to PRRSV (MOI = 50) at 4 °C for 2 h for virus attachment, a temperature shift to 37 °C triggered endocytosis of virions, redistribution of MYH9 to the plasma membrane of PAMs could be detected as early as 5 min, and even increased at 15 min after the temperature shift (Figure S1 and Figure 4A). Meanwhile, co-localization of PRRSV virions and MYH9 was observed as well (Figure 4A). However, after pretreating PRRSV virions with PRA, redistribution of MYH9 to the plasma membrane was much lower (Figure 4A). To further confirm the decreased redistribution of MYH9 to the plasma membrane in PRA-treated cells, total plasma membranes were isolated. As shown in Figure 4B, only a small amount of MYH9 was detected in the plasma membrane fraction of uninfected PAMs for up to 15 min after a temperature shift from 4 °C to 37 °C, whereas MYH9 redistribution increased drastically in PRRSV-infected PAMs after the same temperature shift. However, after preincubation of PRRSV virions with PRA, much less MYH9 was observed in the plasma membrane fraction, while Na^+^/K^+^-ATPase α1 (a ubiquitously expressed membrane protein) was similarly detected across the various groups (Figure 4B). Meanwhile, the total MYH9 level in whole-cell lysates from the various groups of treated cells was unaffected by temperature or PRRSV attachment earlier than 15 min after a temperature switch to 37 °C (Figure 4C), which suggested that increased MYH9 movement to plasma membranes was solely induced by PRRSV attachment rather than alteration of total MYH9 expression levels in PAMs.

To further confirm that reduced plasma membrane redistribution is consistent with reduced PRRSV virion internalization, we evaluated the levels of attached and internalized PRRSV by quantifying PRRSV RNA via qPCR. Based on the result, without triggering endocytosis-mediated viral internalization, the attached virions were similar among groups (Figure 4D). However, if the endocytosis of virion was triggered, PRRSV RNA levels inside PRA-treated PRRSV-infected PAMs or MF4 peptide-treated PRRSV-infected PAMs were significantly lower than that of PRRSV-infected cells without PRA treatment (Figure 4E), which is consisted with the previous result that Mab2-5G2 treatment of PAMs reduced internalized PRRSV virion as well. Taken together, these data indicate that the preincubation of PRRSV virions with PRA as a decoy to disrupt the interaction between GP5 and MYH9 prevents the membrane redistribution of MYH9 thus inhibits internalization of PRRSV virions into permissive cells.

### 3.5. Identification of Key amino acid (aa) Residues Involved in Mab2-5G2 Binding sites within MYH9

**Figure 5:**
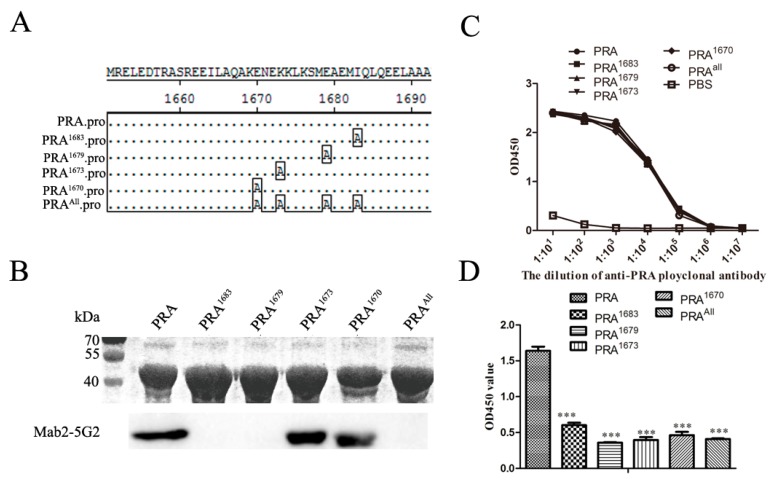
Mutagenesis of predicted Mab-5G2 docking sites on MYH9 affects binding of PRA to Mab2-5G2 and PRRSV virions. (**A**) Schematic illustration of point mutations introduced into the PRA region within the full-length MYH9 protein. (**B**) SDS-PAGE and Western blot analysis to evaluate recombinant protein purity and recognition by Mab2-5G2. (**C**) ELISA analysis to assay the binding PRA mutant proteins with the anti-PRA rabbit serum made by our lab. (**D**) ELISA analysis for quantification of binding between Mab2-5G2 and PRA mutant proteins using Mab2-5G2 as plate-coating protein and anti-PRA rabbit serum for detection of binding between Mab2-5G2 and PRA mutant proteins. ELISA data presented as the mean ± SD. Significant differences between the two groups are marked by “***” (*p* < 0.001).

As the above data suggested that interaction between GP5 and MYH9 plays a key role in PRRSV internalization, we further examined if these predicted amino acids of PRA involved in Mab2-5G2 binding and recognition of MYH9. Therefore, point mutations were introduced at Glu1670, Lys1673, Glu1679, and Ile1683 within the PRA domain to replace wild-type amino acids with alanine (Figure 5A). Expression of all PRA mutants and their reactivity with rabbit anti-PRA polyclonal antibodies were confirmed (Figure 5B). Meanwhile, the reactivity of PRA mutant proteins with Mab2-5G2 was verified, in which PRA mutants (PRA^1683^, PRA^1679^, and PRA^All^) were not recognized by Mab2-5G2, while PRA^1673^ and PRA^1670^ did bind to Mab2-5G2 (Figure 5B).

To determine the binding affinity between PRA mutants and Mab2-5G2, nondenaturing ELISAs of Mab2-5G2 and PRA mutants were conducted. The rabbit anti-PRA polyclonal serum was tested for binding with PRA mutants. As demonstrated in Figure 5C, the binding affinity to anti-PRA serum is similar between wild type PRA and PRA mutants (Figure 5C), while mutations of any potential docking sites (Glu1670, Lys1673, Glu1679, or Ile1683) significantly reduced the binding of Mab2-5G2 to PRA mutants. However, concurrent mutation of all predicted sites did not result in a proportionately greater reduction in Mab2-5G2 binding. Meanwhile, the size-exclusion chromatography (SEC) was conducted to analyze the aggregation of wild-type PRA and PRA mutants (Figure S3); based on the absorbances peaks (mAU 280nm) for wild-type PRA and PRA mutants, no drastically changing was observed for PRA mutants compared to wild-type PRA.

### 3.6. Impaired Binding of Mab2-5G2 to PRA Mutants Correlates with Reduced Inhibition of PRRSV by PRA Mutants

**Figure 6:**
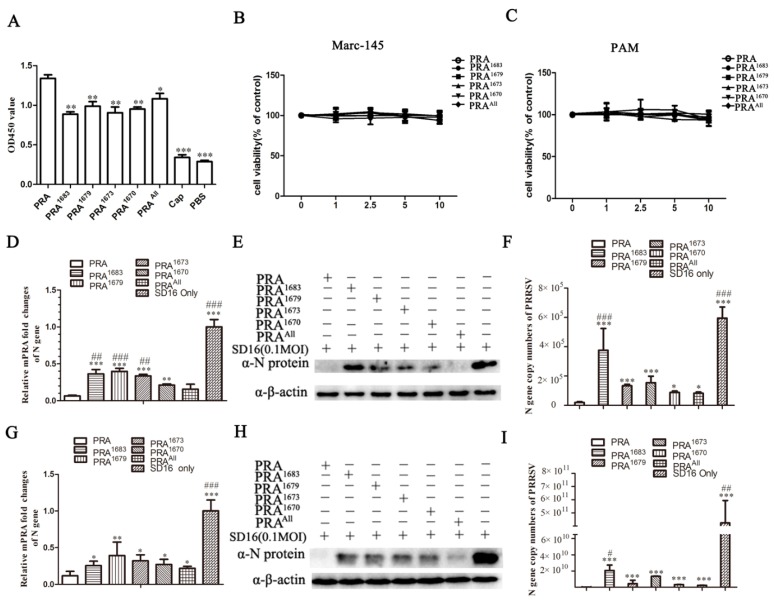
Impaired binding of Mab2-5G2 to PRA mutants correlates with reduced inhibition of PRRSV. (**A**) An ELISA-based virus capture assay performed by coating plates with PRA mutant proteins to capture free PRRSV virions followed by detection of captured PRRSV virions using PRRSV-convalescent swine serum and visualized by HPR-labeled goat anti-swine IgG secondary antibody. Wild-type PRA and recombinant PCV2 Cap protein served as positive control and negative control, respectively. (**B**) Potential cytotoxicity of PRA mutants (PRA^1683^, PRA^1679^, PRA^1673^, PRA^1670^, PRA^all^) against MARC-145 cells was detected with CCK-8 kit. Increasing doses (0,1, 2.5, 5, and 10 µM) of PRA mutants were applied to 80% confluent cells for 24 h and tested for viability via CCK-8 assay respectively. (**C**) Potential cytotoxicity of PRA mutants (PRA^1683^, PRA^1679^, PRA^1673^, PRA^1670^, PRA^all^) against PAM cells was detected with CCK-8 kit. Increasing doses (0,1, 2.5, 5, and 10 µM) of PRA mutants were applied to 80% confluent cells for 24 h and tested for viability via CCK-8 assay, respectively. (**D**) Mutated PRA (5 µM) was pre-mixed with indicated PRRSV SD16 (MOI = 0.1) at 37 °C for 1 h before infecting PAMs. Virus replication was monitored by measuring PRRSV-N protein mRNA levels via qPCR. (**E**) Mutated PRA (5 µM) was pre-mixed with indicated PRRSV SD16 (MOI = 0.1) at 37 °C for 1 h and used to infect PAMs; cells from different treatment groups were harvested 24 h later for Western blot analysis to examine PRRSV-N protein levels. Wild-type PRA was included as positive control. (**F**) Virus RNA copies in supernatants of PRRSV-infected PAMs in different groups were harvested and determined by absolute quantification via qPCR. (**G**) Inhibitory activity of mutated PRA toward infection by the PRRSV-SD16 strain was determined in MARC-145 cells. Mutated PRA (or positive control PRA protein) (5 µM) was pre-mixed with indicated PRRSV SD16 (MOI = 0.1) at 37 °C for 1 h before infecting MARC-145. Virus replication was monitored by measuring PRRSV N protein mRNA levels via qPCR. (**H**) Mutated PRA (5 µM) was pre-mixed with indicated PRRSV SD16 (MOI = 0.1) at 37 °C for 1 h and used to infect MARC-145 cells; cells from different treatment groups were harvested 24 h later for Western blot analysis to examine PRRSV-N protein levels. (**I**) Virus RNA copies in supernatants harvested from indicated groups of MARC-145 were determined by absolute quantification via qPCR. Experiments were repeated at least three times. All data are expressed as mean ± SD and were subjected to Student’s *t*-test. Significant differences between SD16 only groups to PRA and PRA mutants groups are marked by “*” (*p* < 0.05), “**” (*p* < 0.01), and “***” (*p* < 0.001). Significant differences between PRA^All^ groups to PRA and PRA mutants groups are marked by “#” (*p* < 0.05), “##” (*p* < 0.01), and “###” (*p* < 0.001).

To identify which key amino acid residue sites located in MYH9 played a crucial role in PRRSV internalization, the recombinant PRA protein, as well as PRA mutants, were used for competitive binding for PRRSV virion and PRRSV internalization blocking assay. Theoretically, mutagenesis of above amino acid residues in PRA would impair binding of PRA mutants to virus, which reduce the inhibition effect of PRRSV by corresponding PRA mutants. To verify this, we first tested the PRA mutants developed here for an ELISA-based virus binding assay described before [35]. The results showed that all PRA mutants had significantly lower virus-binding activity than wild-type PRA (Figure 6A). However, the PRA mutant with substitutions of all four amino acids exhibited binding affinity to virions that were higher than that of single site-mutated PRA mutants, although antibody binding was still significantly lower than that of wild-type PRA (Figure 6A). As recombinant PRA inhibited PRRSV infection in permissive cells via its interaction with virion GP5 [35], we propose that PRA proteins bearing mutation sites predicted to be involved in docking with Mab2-5G2 should exhibit impaired inhibition of PRRSV replication in susceptible cells. Based on a cell viability assay using MARC-145 cells and PAMs (Figure 6B,C), 5µM of each PRA mutant was selected for further evaluation.

As shown in Figure 6D, except for PRA^All^, all PRA mutants bearing single mutations demonstrated increased PRRSV-SD16 infection by measuring RNA expression level of PRRSV-N as compared to wild-type PRA in MARC-145 cells, which was consistent with results from N protein-level (Figure 6E) and absolute quantification of viral RNA copies in cell supernatants (Figure 6F). It is notable that among PRA mutants, PRA^1683^ displayed the minimum PRRSV inhibition compared with other PRA mutants and wild-type PRA (Figure 6D–F), suggested that Ile in aa 1683 might be the key aa residue involved in the interaction between PRA and GP5 protein of virion. In contrast, the inhibition activity of PRRSV infection in MARC-145 cells by addition of PRA^All^ was comparable to that of wild-type PRA (Figure 6D,E), while absolute viral RNA copies from supernatants of PRA^All^ treated group demonstrated slight elevation (Figure 6F). Conversely, PRRSV inhibition of PRA mutants evaluated in PAMs demonstrated a similar trend as that of MARC-145 cells (Figure 6G–I). Taken together, these data documented that single-point mutations within the predicted Mab2-5G2 docking site affected PRRSV replication efficiency, a result which may be partially due to reduced binding affinity between PRA mutants and PRRSV virions. Therefore, those data implied that MYH9 bearing single aa mutation described above may confer PRRSV resistance in permissive cells due to the reduced interaction between GP5 and MYH9 but require further investigation.

### 3.7. Porcine Cell Line Bearing Point Mutation in E1670A in MYH9 Demonstrated Reduced Susceptibility for PRRSV Infection

**Figure 7:**
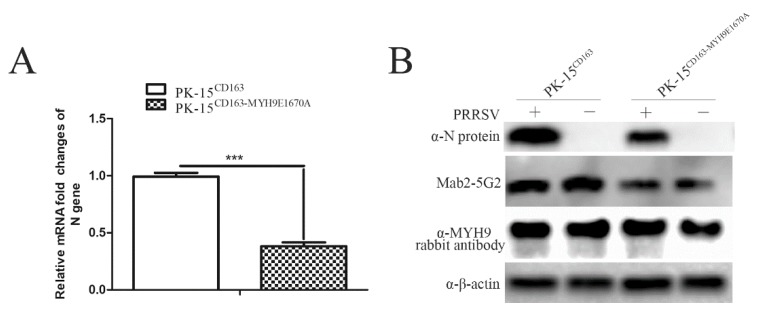
E1670A mutation of MYH9 in permissive cells confer partial resistance to PRRSV infection. (**A**) PRRSV permissive PK-15^CD163^ cells and PK-15^CD163-MYH9E1670A^ were infected by PRRSV at 0.1 MOI for 24 h then harvested using TRIzol for relative quantification of viral RNA by qPCR. The data are expressed as mean ± SD and were subjected to Student’s *t*-test. Significant differences between the two groups are marked by “***” (*p* < 0.001). (**B**) PRRSV permissive PK-15^CD163^ cells and PK-15^CD163-MYH9E1670A^ were infected by PRRSV at 0.1 MOI for 24 h or left uninfected. Samples were harvested for Western blot analysis to examine PRRSV-N protein levels.

To validate the speculation that key amino acid residue sites in MYH9 identified above play a crucial role to determine PRRSV infection in permissive cells, we conducted a preliminary experiment to introduce the aa mutant into E1670 position of MYH9 using CRISPR/CAS9 in PK-15 cells bearing CD163, which is susceptible for PRRSV infection with swine origin. After confirming the successful generation of PK-15^CD163-MYH9E1670A^ by DNA sequencing for corresponding mRNA, we infected wild-type PK-15CD163 cells and PK-15^CD163-MYH9E1670A^ by PRRSV at 0.1 MOI. Based on data, a more than 50% reduction of PRRSV-N mRNA level is observed in PK-15^CD163-MYH9E1670A^ compared to wild-type PK-15^CD163^ cells (Figure 7A). Meanwhile, the expression of PRRSV-N protein in both cells line was examined as well. As demonstrated in Figure 7B, there is significant inhibition of PRRSV-N proteins, which is consisted of qPCR results. Moreover, a reduced reactivity of Mab2-5G2 to MYH9 with E1670A mutation was observed as well, while the wild-type MYH9 or MYH9^E1670A^ level appears to be similar between these two cell lines if probed with anti-MYH9 antibodies. Taken together, these preliminary data indicated that aa mutation in sites determine interaction between GP5 and MYH9 could confer partial PRRSV resistance in permissive cells.

## 4. Discussion

Generally, MYH9 functions as a motor protein involved in cell migration, integrin-mediated adhesion, epithelial cell polarization, cell-cell adhesion, and morphogenesis [42]. In recent years, several studies have implicated MYH9 as a potential receptor involved in infection by viruses such as HSV-1, SFTSV, EBV, and PRRSV [21,31,32,33]. Recent studies have shown MYH9 to be indispensable for PRRSV internalization and intercellular spread in permissive cells [21,41]. As a cytoplasmic protein, there is little plasma membrane distribution of MYH9 under normal conditions. However, data gained from HSV, EBV, and PRRSV infections suggest that redistribution of MYH9 from the cytoplasm to plasma membrane appears to be a common step required for virus internalization after virus attachment to susceptible cells [21,31,32,33]. Moreover, interactions between MYH9 and viral proteins such as PRRSV-GP5 or gB of HSV-1 have been observed, with blockage of MYH9 binding with specific antibody resulting in inhibition of virus infection in those systems [21,31].

In this study, we demonstrated that Mab2-5G2 blocked PRRSV infection of PAMs, suggesting that Mab2-5G2 may serve as a “competitive binding partner” that blocks the interaction between GP5 and MYH9 during PRRSV internalization by permissive cells. Meanwhile, truncations of full-length MYH9 protein helped to localize the minimal domain recognized by Mab2-5G2 more precisely to the MF fragment within the PRA region (aa1651–1716 of full length MYH9). Subsequent work using synthetic peptides of the PRA MF fragment suggested that aa1668–1696 encompassed the major epitope needed for Mab2-5G2 binding, which was confirmed using dot blot analysis. Based on these findings, we predicted the 3D structure for the Mab2-5G2 Fab fragment bound to PRA via molecular modeling of Mab2-5G2 using known structures of highly homologous immunoglobulins and circular dichroism spectroscopy results for PRA. Based on the predicted model, several potential docking sites required for Mab2-5G2 binding to PRA region were explored and validated by mutagenesis. Based on our data, it appears that PRA bearing mutations of predicted docking sites demonstrated reduced inhibition for PRRSV replication in vitro. These results may be partially due to impaired binding between PRA and PRRSV virions via GP5 protein, which implied full-length MYH9 bearing mutations of the abovementioned sites may not effectively mediate PRRSV internalization. To validate this expectation, E1670A mutation was introduced to MYH9 of PRRSV permissive PK-15^CD163^ cells, and our data suggested that cells bearing E1670A mutation in MYH9 confer partial resistance for PRRSV infection in vitro compared to wild-type PK-15^CD163^.

On the contrary, our preliminary data suggested that Mab2-5G2 demonstrated little antiviral activity against the PRRSV-1 strain GZ11 [43]. Since PRRSV-1 and PRRSV-2 only share 60% homology at the genome level, little attention has been paid to determine if different receptor preferences exist between PRRSV-1 and PRRSV-2, especially since several receptors are involved in PRRSV infection. As an example, available data suggests porcine CD163 (pCD163) acts as a fusion receptor for PRRSV infection [44,45]. The extracellular region of CD163 is composed of nine scavenger receptor cysteine-rich (SRCR) domains (SRCR1-9) [46]. Truncation assays have demonstrated that the SRCR5 domain of pCD163 is required for PRRSV infection [46], results further supported by the fact that genome-edited pigs bearing CD163 lacking SRCR5 were resistant to viruses of both PRRSV-1 and PRRSV-2 [47]. However, it is notable that replacement of the SRCR5 domain of pCD163 with the SRCR5 domain of the human CD163-like homolog (CD163Li) only conferred resistance to strains of PRRSV-1, but not to PRRSV-2 [48], implying different preferred receptor utilization between PRRSV-1 and PRRSV-2 strains. Moreover, macrophages play a pivotal role in the host immune system as macrophage subset is specialized in function and reflected by the expression of different receptors such as complement receptors, Fc-receptors, adhesion molecules, and receptors for various soluble mediators [49]. Another issue that is less investigated for PRRSV infection is the role of antibody-dependent enhancement (ADE) of infection as previous report demonstrated that non-neutralizing antibody (non-NA) was responsible for ADE during PRRSV infection in PAMs that involves various Fc receptors [50,51,52,53]. However, the relationship between CD163, MYH9, and other receptors such as Fc receptors, as well as their corresponding contribution to PRRSV attachment and internalization into permissive cells remains elusive, it is possible that a different receptor preference exists between the two genotypes of PRRSV. On the one hand, replacement of SRCR5 of pCD163 with the corresponding domain of human CD163Li can confer resistance to PRRSV-1 but not PRRSV-2 infection of permissive cells, suggesting that PRRSV-1 infection, therefore, may be less reliant on other receptors but more CD163-restricted than is PRRSV-2. On the other hand, data presented in this study implies that PRRSV-2 infection may require cooperation of different receptors (such as MYH9) to reach maximum infectivity, while PRRSV-2 maintains a CD163-determined cell tropism, which is partially supported by our previous observation that co-expression of MYH9 and CD163 was required to render COS7 cells (a PRRSV non-permissive cell line) susceptible to PRRSV-2 infection, with sole expression of CD163 insufficient to support PRRSV-2 replication [21].

In conclusion, our study indicates that Mab2-5G2 could serve as a novel competitive inhibitor for PRRSV entry via mimic PRRSV-GP5 proteins to interact with MYH9. Moreover, interruption of interaction between PRRSV-GP5 and MYH9 blocks PRRSV internalization but not PRRSV attachment to susceptible cells. Furthermore, homology modeling of Mab2-5G2 and interacting counterpart domain with MYH9 underlined several key amino acid residues as potential sites required for PRRSV virion internalization and one of such aa residues is partially validated in PRRSV permissive cells line using CRISPR/CAS9 technology. These results should increase our understanding of PRRSV entry, as well as guide the development of novel antiviral strategies against PRRSV infection.

## Figures and Tables

**Table 1:** Primers used to construct PRA (C-terminal domain of MYH9), truncated PRA, and site-directed PRA mutants and qPCR.

Genes Name	(Sense 5′–3′)
PRA-F	ACGCGTCGACATGCGGGAGCTGGAG
PRA-R	CGGGATCCTCATTGTACTTCAGAGT
PRA^1651-1716^F	ATGTCGACATGCGGGAGCTGGAGGACAC
PRA^1651-1716^-R	ATTCTAGATTTGCCGCTGCTGTTGGCAA
PRA^1714-1960^-F	CGGGATCCATGGGCAAAGGGGCG
PRA^1714-1960^-R	ATTCTAGATTATTCGGCAGGTTT
PRA^1670^-F	GCACAGGCCAAGGCAAACGAGAAGAAGCTGAAAAGCATGGAGGCC
PRA^1670^-R	CTTCTTCTCGTTTGCCTTGGCCTGTGCCAGGATCTCCTCGCGGGA
PRA^1673^-F	AAGGAGAACGAGGCAAAGCTGAAAAGCATGGAGGCCGAGATGATC
PRA^1673^-R	GCTTTTCAGCTTTGCCTCGTTCTCCTTGGCCTGTGCCAGGATCTC
PRA^1679^-F	CTGAAAAGCATGGCAGCCGAGATGATCCAGCTGCAGGAGGAGCTG
PRA^1679^-R	GATCATCTCGGCTGCCATGCTTTTCAGCTTCTTCTCGTTCTCCTT
PRA^1683^-F	GAGGCCGAGATGGCACAGCTGCAGGAGGAGCTGGCCGCCGCCGA
PRA^1683^-R	CTCCTGCAGCTGTGCCATCTCGGCCTCCATGCTTTTCAGCTTCTT
ORF-7-F	ATGCCAAATAACAACGGCAAGCAGC
ORF-7-R	TCATGCTGAGGGTGATGCTGTG
GAPDH-F	CCTTCCGTGTCCCTACTGCCAAC
GAPDH-R	GACGCCTGCTTCACCACCTTCT

## Supplementary Materials

The following are available online at https://www.mdpi.com/1999-4915/12/1/40/s1, Figure S1: Nucleotide and deduced amino acid sequenced of VL and VH of Mab2-5G2, Figure S2: Redistribution of MYH9 to the plasma membrane is involved in internalization of PRRSV virions, Figure S3: The size-exclusion chromatography (SEC) for aggregation of wild-type PRA and PRA mutants.
