# Supplementary material for: MYH9 Key Amino Acid Residues Identified by the Anti-Idiotypic Antibody to Porcine Reproductive and Respiratory Syndrome Virus Glycoprotein 5 Involve in the Virus Internalization by Porcine Alveolar Macrophages
Source: Viruses. 2019 Dec 29;12(1):40. doi: 10.3390/v12010040 (PMC7019770; doi:10.3390/v12010040)
Supplement: Supplementary file 1 [file viruses-12-00040-s001.pdf]

V_L

```
FR-L1
GACATCAAGATGACCCAGTCTCCATCCTCCATGAATGCATCGCTGGGAGAGAGAGTCACT
 D  I  K  M  T  Q  S  P  S  S  M  N  A  S  L  G  E  R  V  T
                   CDR-L1                                FR-L2
ATCACTTGCAAGGCGAGTCAGGACATTAAAAGCTATTTAAGCTGGTACCAGCAGAAACCA
 I  T  C  K  A  S  Q  D  I  K  S  Y  L  S  W  Y  Q  Q  K  P
                              CDR-L2                       FR-L3
TGGAAATCTCCTAAGACCCTGATCTTTTATGCAACAAGCTTGGCCGATGGGGTCCCATCA
 W  K  S  P  K  T  L  I  F  Y  A  T  S  L  A  D  G  V  P  S

AGATTCAGTGGCAGTGGATCTGGTCAAGATTATTCTCTAACCATCAGCAGCCTGGAGTCT
 R  F  S  G  S  G  S  G  Q  D  Y  S  L  T  I  S  S  L  E  S
                           CDR-L3
GACGATACAGCAACTTATTACTGTCTACAGCATGGTGAGAGCCCTCTCACGTTCGGTGCT
 D  D  T  A  T  Y  Y  C  L  Q  H  G  E  S  P  L  T  F  G  A

GGGACCAAGCTGGAGCTGAAACGGGCTGATGCTGCTGCACCAACTGTATCCATCTTC
 G  T  K  L  E  L  K  R  A  D  A  A  A  P  T  V  S  I  F
```

V_H

```
FR-H1
GTGCAGCTTCAGGAGTCGGGACCTGGCCTGGTGAAACCTTCTCAGTCTCTGTCCCTCACC
 V  Q  L  Q  E  S  G  P  G  L  V  K  P  S  Q  S  L  S  L  T
            CDR-H1                                   FR-H2
TGCACTGTCACTGGCTACTCAATCACCAGTGATTATGCCTGGAACTGGATCCGGCAGTTT
 C  T  V  T  G  Y  S  I  T  S  D  Y  A  W  N  W  I  R  Q  F
                              CDR-H2
CCAGGAAACAAACTGGAGTGGATGGGCTACATAAGCTACAGTGGTAACACTCGCTATAAC
 P  G  N  K  L  E  W  M  G  Y  I  S  Y  S  G  N  T  R  Y  N
FR-H3
CCATCTCTCAAAAGTCGAATCTCGATCACTCGAGACACATCCAAGAACCAGTTCTTCCTG
 P  S  L  K  S  R  I  S  I  T  R  D  T  S  K  N  Q  F  F  L
                                                 CDR-H3
CAGTTGAATTCTGTGACTACTGAGGACACAGCCACATATTACTGTGCAAGATCGGGTTTC
 Q  L  N  S  V  T  T  E  D  T  A  T  Y  Y  C  A  R  S  G  F

TATAGGTACGACGGCTGGTTTGCTTACTGGGGCCAAGGGACTCTGGTCACTGTCTCTGCA
 Y  R  Y  D  G  W  F  A  Y  W  G  Q  G  T  L  V  T  V  S  A
```

Figure S1. Nucleotide and deduced amino acid sequenced of VL and VH of Mab2-5G2.

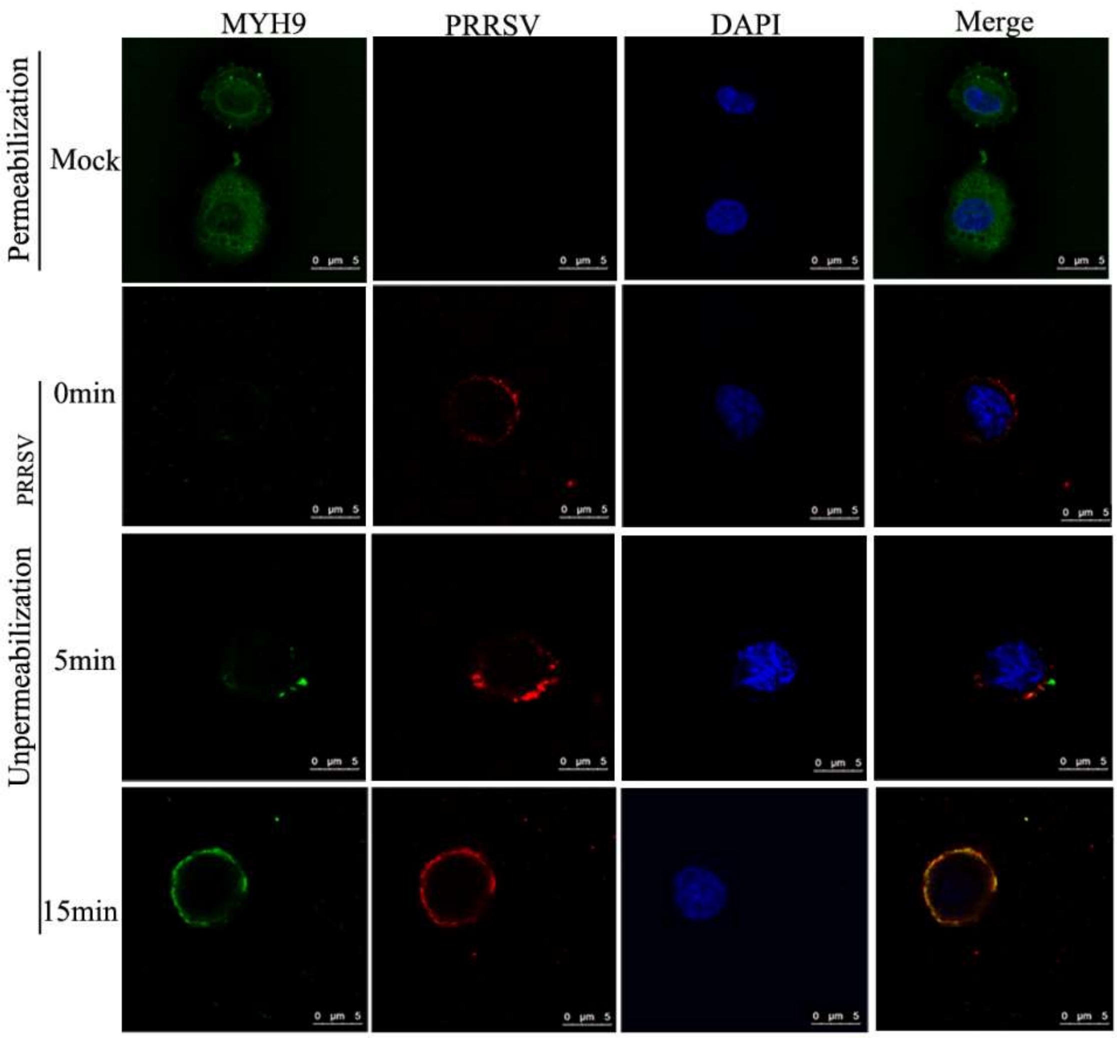

Figure S2. Redistribution of MYH9 to the plasma membrane is involved in internalization of PRRSV virions. Confocal microscopy of MYH9 redistribution from cytoplasm to membranes of PAMs receiving indicated treatments. PAMs were incubated with PRRSV (50MOI) at 4°C for 2 h before shifting to 37°C. Cells with indicated time point after temperature switch was fixed and stained with PRRSV positive serum and Mab2-5G2 to visualize viral particle and plasma membrane distribution of MYH9 without membrane permeabilization. Also including normal PAM cell as control.

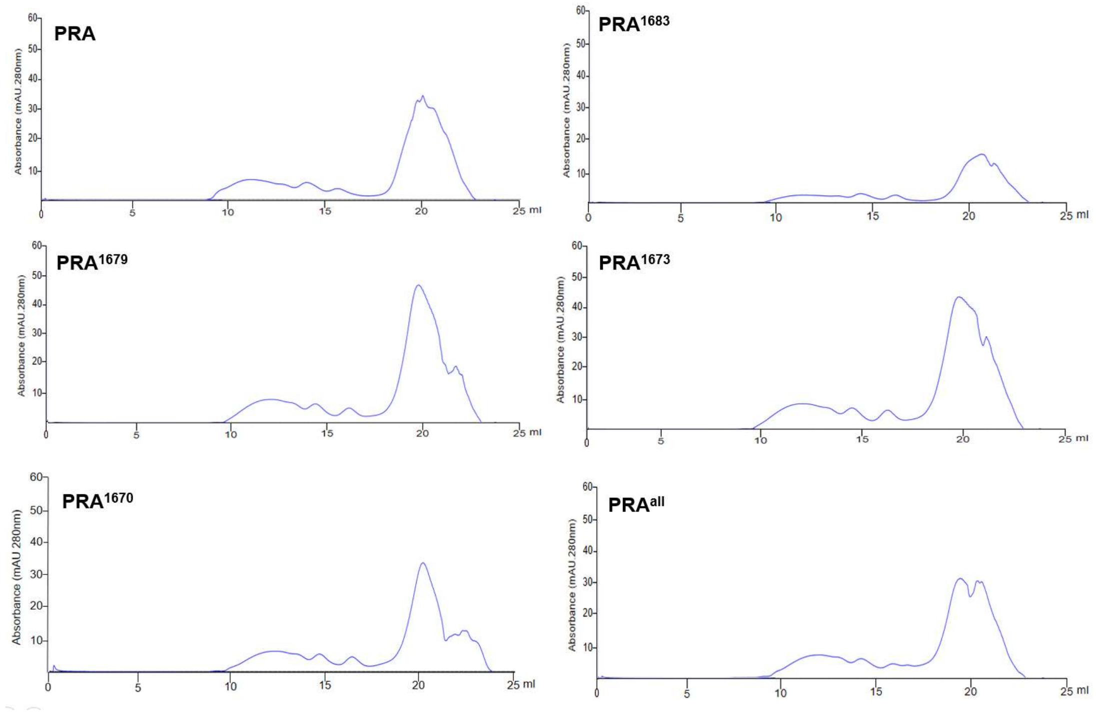

Figure S3. The Size-exclusion chromatography (SEC) for aggregation of wild-type PRA and PRA mutants.
